# A long hypoxia-inducible factor 3 isoform 2 is a transcription activator that regulates erythropoietin

**DOI:** 10.1007/s00018-019-03387-9

**Published:** 2019-11-25

**Authors:** Jussi-Pekka Tolonen, Minna Heikkilä, Marjo Malinen, Hang-Mao Lee, Jorma J. Palvimo, Gong-Hong Wei, Johanna Myllyharju

**Affiliations:** 1 Oulu Center for Cell–Matrix Research, University of Oulu, PO Box 5400, 90014 Oulu, Finland; 2 Biocenter Oulu and Faculty of Biochemistry and Molecular Medicine, University of Oulu, 90014 Oulu, Finland; 3 Department of Environmental and Biological Sciences, University of Eastern Finland, 80100 Joensuu, Finland; 4 Institute of Biomedicine, University of Eastern Finland, 70211 Kuopio, Finland

**Keywords:** Hypoxia response, Hypoxia-inducible factor 3 isoform, Transcription activator, Erythropoietin, Chromatin immunoprecipitation, Hypoxia response element

## Abstract

Hypoxia-inducible factor (HIF), an αβ dimer, is the master regulator of oxygen homeostasis with hundreds of hypoxia-inducible target genes. Three HIF isoforms differing in the oxygen-sensitive α subunit exist in vertebrates. While HIF-1 and HIF-2 are known transcription activators, HIF-3 has been considered a negative regulator of the hypoxia response pathway. However, the human *HIF3A* mRNA is subject to complex alternative splicing. It was recently shown that the long HIF-3α variants can form αβ dimers that possess transactivation capacity. Here, we show that overexpression of the long HIF-3α2 variant induces the expression of a subset of genes, including the erythropoietin (*EPO*) gene, while simultaneous downregulation of all HIF-3α variants by siRNA targeting a shared *HIF3A* region leads to downregulation of *EPO* and additional genes. EPO mRNA and protein levels correlated with *HIF3A* silencing and HIF-3α2 overexpression. Chromatin immunoprecipitation analyses showed that HIF-3α2 binding associated with canonical hypoxia response elements in the promoter regions of *EPO*. Luciferase reporter assays showed that the identified HIF-3α2 chromatin-binding regions were sufficient to promote transcription by all three HIF-α isoforms. Based on these data, HIF-3α2 is a transcription activator that directly regulates *EPO* expression.

## Introduction

Oxygen-dependent organisms have developed elaborate means to maintain appropriate intracellular oxygen levels for the physicochemical reactions that occur within cells. The master regulators of oxygen homeostasis are the heterodimeric hypoxia-inducible factors (HIFs), present in some form in all metazoan species studied so far [1, 2]. When intracellular oxygen tension decreases below a typical concentration, the HIFs initiate and control graded mechanisms to reduce oxygen consumption and to increase oxygen availability via oxygen-dependent regulation of a genetic hypoxia response pathway [3, 4].

Three HIF-α subunit isoforms (HIF-1α, HIF-2, and HIF-3α) have been identified in vertebrates, encoded by three separate genes (*HIF1A*, *EPAS1,* and *HIF3A*), with *HIF3A* mRNA being subject to diverse alternative splicing [5–10]. HIF-1α, HIF-2α, and some HIF-3α variants contain basic helix–loop–helix–PAS domains, which facilitate heterodimerization with the HIF-β subunit, encoded by the *ARNT* gene, and binding to DNA [11]. Towards their C-terminus, the HIF-α protein also possess an oxygen-dependent degradation domain (ODDD), which accounts for their oxygen-dependent regulation. HIF-1α and HIF-2α contain two transactivation domains (NTAD and CTAD), whereas the long *HIF3A* splicing variants contain only the NTAD [12].

The HIFs bind to specific hypoxia-responsive elements (HREs) with a canonical core sequence (5′–RCGTG-3′) [13]. According to genome-wide chromatin immunoprecipitation sequencing (ChIP-seq) studies, approximately 40% and 20% of HREs for HIF–1 and HIF–2, respectively, reside in promoter regions within a 2.5-kb range from transcription initiation sites (TSS) [14]. HIF–1 appears to bind HREs in promoter regions, while HIF-2 binds more distant regions such as enhancers [15]. Most importantly, however, HIF-1 and HIF-2 do not compete for the same binding sites [15]. The HIF-3 αβ dimer has been shown to recognize the canonical HRE [16], but the genome-wide binding of the human HIF-3 and its consequences have not been characterized.

Erythropoietin (EPO), the main regulator of red blood cell production, is a classic hypoxia-inducible gene mainly targeted by HIF-2 [17]. It is produced in the liver during early development and later in the kidney [18]. A 256-bp liver inducibility element (LIE) has been identified immediately 3′ to the gene in Hep3B cells, whereas a negative regulatory element (NRE) lies 4–6 kb upstream of the gene [19–22]. A kidney inducibility element (KIE) lies even farther upstream (9.5 to 14 kb), regulating *EPO* transcription in peritubular interstitial fibroblasts [17, 21]. The single HRE found in the LIE is crucial for maximal *EPO* expression and is probably bound only by HIF-2 in accordance with HIF-2 binding to enhancers [15, 17, 22–24]. Although some candidates for the kidney-specific HRE have been identified in vitro and in vivo, no definite consensus exists [24–26]. Of note, *HIF1A* knockdown does not suppress *EPO* expression in the three cell lines studied so far, namely, Hep3B, Kelly, and cortical astrocytes [23, 27].

Studies of the HIF pathway have thus far focused mainly on HIF-1 and HIF-2, leaving HIF-3 a relatively unknown regulator of the hypoxia response. Previous experiments conducted in mice suggested that a short splice variant of *Hif3a*, the inhibitory PAS domain containing protein (IPAS), acts as a dominant negative inhibitor of the hypoxia response by forming inactive complexes with HIF-1α and HIF-2α [5–7, 9, 28]. The short human *HIF3A* splice variant, HIF-3α4, inhibits the hypoxia response in a similar dominant negative manner [10, 12, 29]. However, more recent studies have suggested that each HIF-3α variant may perform manifestly different roles and that the long HIF-3 variants possess transactivation activity [9, 12, 30, 31]. Furthermore, we have previously shown that simultaneous in vitro knockdown of all human *HIF3A* splice variants results in the downregulation of several hypoxia-inducible genes including *EPO* [12]. Similar to other transcription factors with dominant negative and transactivating splice variants, such as the IKAROS family zinc finger 1 [32], HIF-3 may thus be a hypoxia-inducible transcription factor with a dual role.

To test our hypothesis that human HIF-3 can induce the transcription of certain hypoxia-inducible genes, we carried out a cDNA microarray screen of hypoxia-dependent HIF-3 target genes in Hep3B cells. Under hypoxia, an overexpression of the long HIF-3α2 splice variant resulted in over twofold upregulation of eight genes, including *EPO*. HIF-3 clearly contributed to EPO signaling as overexpression of HIF-3α2 in two cell lines capable of endogenous EPO production, namely, Hep3B and the SK-N-AS neuroblastoma cell line, and siRNA knockdown of all *HIF3A* variants in the SK-N-AS cells resulted in significant changes in EPO mRNA and protein levels that are in line with the hypothesis that HIF-3 is a transcription activator. Our ChIP data suggest that HIF-3 binds its target genes via the canonical HRE. The HIF-3-binding regions are sufficient to drive the transcription of luciferase reporter genes when co-transfected with one of the HIF-α isoforms and HIF-β. These data indicate that at least one of the long HIF-3α variants is a transcription activator involved in erythropoietin signaling by binding directly on *EPO* and inducing its transcription.

## Materials and methods

### Cell culture

Hep3B hepatoma cells were cultured in Earle’s minimum essential medium (Sigma, USA), ChoK1 cells were cultured in Dulbecco’s minimum essential medium (Biochrom AG, Germany) with 0.375% sodium bicarbonate (Sigma) and SK-N-AS neuroblastoma cells were cultured in RPMI 1640 (Gibco, USA). The culture media for Hep3B and ChoK1 cells were supplemented with 0.1 mM non-essential amino acids (Sigma), 1 mM sodium puryvate (Sigma), 10% fetal bovine serum (HyClone, USA), 2 mM _l_-glutamine (Sigma), and 100 U/ml penicillin with 0.1 mg/ml streptomycin (Gibco), while the SK-N-AS culture medium was supplemented with 10% fetal bovine serum (Sigma) and 100 U/ml penicillin with 0.1 mg/ml streptomycin (Gibco). Cell culture under hypoxic conditions (1% O_2_, 5% CO_2,_ and 94% N_2_) was performed in the Invivo_2_ Hypoxia Workstation 400 (Ruskinn Technologies, UK) for cDNA microarray studies, and the Sci-Tive-N (Baker Ruskinn, UK) hypoxia station for all other experiments. As the Hep3B cells express *EPO* and other endogenous human hypoxia-inducible genes, they were chosen for the ChIP, cDNA microarray and functional experiments. The ChoK1 cell line was used as a host for the luciferase reporter assay due to its high transfection rate. The SK-N-AS cells that also express *EPO* were used to confirm results obtained in Hep3B cells.

### Expression plasmids and preparation of the HIF-3α antibody

The following expression plasmids described previously were used in this study: pcDNA3.1/Zeo(-)-V5-HisA, pEGFP-N1, full-length untagged human HIF-1α, HIF-2α, and HIF-β, and untagged as well as C-terminal V5-tagged human HIF-3α2 [10, 12].

To generate luciferase reporter constructs for HIF-3α2 binding sites identified by ChIP-seq in *EPO*, angiopoietin-like-4 (*ANGPTL4*) and Histone Cluster 1 H2B Family Member K (*HIST1H2BK*) genes, the DNA for the binding sites was amplified and cloned into the pGL4.75 vector (Promega, USA). To study the dependency of HIF-1α and HIF-3α2 binding on the canonical HRE sequences (5′-RCGTG-3′), all such sites found within the HIF-3α2 *HIST1H2BK* binding site on the forward and reverse strands were mutated to 5′-ATTTA-3′ (denoted mutHIST1H2BK) using the QuickChangeXL II site-directed mutagenesis kit (Stratagene, USA) according to manufacturer’s instructions. Mutagenesis primers are listed in Table 1.

**Table 1:** Sequences of the primers used in cloning, mutagenesis, RNA interference, qPCR analyses, ChIP studies, and luciferase reporter experiments

Gene	Use	Primer ID	Sequence (5′ → 3′)
*ACTB*	qPCR	b-ActinFw	TGTGGCATCCACGAAACTAC
	qPCR	b-ActinRv	TCATACTCCTGCTTGCTGATCC
*ANGPTL4*	ChIP-qPCR	ANGPTL4_cQ_F	AAGTGTATGAGTGGCAGCCT
	ChIP-qPCR	ANGPTL4_cQ_R	AACTTGCACCGATCTCCTCT
	Cloning	ANGPTL4_B_F	GCGAGATCTCACGGTTCGTAGAGGAAGGC
	Cloning	ANGPTL4_B_R	GCGAAGCTTCCCACTCCTGTCCATACCCT
*EIF5A*	ChIP-qPCR	EIF5A_cQ_F	TGGAGATGGGTAGGGTGTGT
	ChIP-qPCR	EIF5A_cQ_R	GACCAACCAAGCAGCCCTAT
*EPAS1*	qPCR	Q_HIF2a_F	CCCAGATCCACCATTACAT
	qPCR	Q_HIF2a_R	ACTCCAGCTGTCGCTTCA
*EPO*	qPCR	EPO_RT_F	CTCCGAACAATCACTGCT
	qPCR	EPO_RT_R	GGTCATCTGTCCCCTGTCCT
*(Control)*	ChIP-qPCR	EPO_ctrl_cQ_F	GGAAGGCAATTTTGTGTGCG
*(Control)*	ChIP-qPCR	EPO_ctrl_cQ_R	CCAAGCACCAGAAACTCACC
	ChIP-qPCR	EPO_cQ_F	CCAGTGGAGAGGAAGCTGAT
	ChIP-qPCR	EPO_cQ_R	CTTCCTTCATCCCCACGTCT
	Cloning	EPO-1_F	GCGGAGCTCGGATTGTGGGAAGGGAGACC
	Cloning	EPO-1_R	GCGCTCGAGATAGCCGGGGCGCTAAATC
*EPOR*	ChIP-qPCR	EPOR_cQ_F	TAGGCAGCGAACACCAGAAG
	ChIP-qPCR	EPOR_cQ_R	TCACACACACACACAAGGCT
*HIF1A*	qPCR	Q_HIF1a_F	CTAGCTTTGCAGAATGCTCAG
	qPCR	Q_HIF1a_R	GTAGTAGCTGCATGATCGTCTG
*HIF3A*	RNAi	siHIF3A_a	UAACAGGGCAGUAUCGCUU
	RNAi	siHIF3A_b	CGACAGGAUUGCAGAAGUG
	RNAi	siHIF3A_c	GCAAGAGCAUCCACACCUU
	RNAi	siHIF3A_d	GAACUGCUCUGGACAUAUG
	RNAi	siHIF3A_e	SR312024B, sequence not available
	qPCR	Q_HIF3a_all_F	CCCCACGGAGCGGTGCTTCT
	qPCR	Q_HIF3a_all_R	AGTCTGCGCAGGTGGCTTGT
*HIST1H2BK*	qPCR	HIST1H2BK_Q_F	TGCTGCTCGTCTCAGGCTCGT
	qPCR	HIST1H2BK_Q_R	CTCTCCTTGCGGCTGCGCTT
	ChIP-qPCR	HIST1H2BK_cQ_F	GGGCCCCTAAGCTTTCAACA
	ChIP-qPCR	HIST1H2BK_cQ_R	GGCTCTTCTGGCCTTGGAAA
	Cloning	HIST1H2BK_R	GCGGAGCTCCGGCGTCGAGTTAATCTTGT
	Cloning	HIST1H2BK_R	GCGCTCGAGTCCGGTTTTCAGTCTGGTCC
	Mutagenesis	HIST1H2BK_HRE1_F	AAGACGGTCACCGCCATGGTAAATGTCTACGCGCTCAAGCGCC
	Mutagenesis	HIST1H2BK_HRE1_R	GGCGCTTGAGCGCGTAGACATTTACCATGGCGGTGACCGTCTT
	Mutagenesis	HIST1H2BK_HRE2_F	GCCGTGACCTACACGGAGTAAATCAAGCGCAAGACGGTCAC
	Mutagenesis	HIST1H2BK_HRE2_R	GTGACCGTCTTGCGCTTGATTTACTCCGTGTAGGTCACGGC
	Mutagenesis	HIST1H2BK_HRE3_F	TGTTGAAGGTGTTCCTGGAGATAAATATCCGGGACGCCGTGACCTACAC
	Mutagenesis	HIST1H2BK_HRE3_R	GTGTAGGTCACGGCGTCCCGGATATTTATCTCCAGGAACACCTTCAACA
	Mutagenesis	HIST1H2BK_HRE4_F	TGCTCGCCGCGGCGTAAATAAGCGCATTTCTGGCCTCATCTATGAG
	Mutagenesis	HIST1H2BK_HRE4_R	CTCATAGATGAGGCCAGAAATGCGCTTATTTACGCCGCGGCGAGCA
*HPRT1*	qPCR	hHprt_F	CCTGGCGTCGTGATTAGTGAT
	qPCR	hHprt_R	AGACGTTCAGTCCTGTCCATAA
*PMB6*	qPCR	hPMB6qfor	AGCGACACCACAAAGAGTTCA
	qPCR	hPMB6qrev	GCTGATGCTCCTGTAAGACTTGA
*PSMD5*	ChIP-qPCR	PSMD5_cQ_F	AATCTTGATCCTGGGCCAGC
	ChIP-qPCR	PSMD5_cQ_R	GCGCACGTCCCTATTACTCA
*PTX3*	qPCR	hPTXqfor	CATCTCCTTGCGATTCTGTTTTG
	qPCR	hPTXqrev	CCATTCCGAGTGCTCCTGA
*SLC6A14*	qPCR	hSLC6A14qfor	ACCGTGGTAACTGGTCCAAAA
	qPCR	hSLC6A14qrev	CGCCTCCACCATTGCTGTAG
*TBP*	qPCR	TBP_Q_F	GAATATAATCCCAAGCGGTTTG
	qPCR	TBP_Q_R	ACTTCACATCACAGCTCCCC
*TMEM27*	qPCR	hTMEM27qfor	CTGGTGACTGCCATTCATGCT
	qPCR	hTMEM27qrev	CCATCGCTTTGAAGAGGTATTCT

To produce the HIF-3α2 antibody used in the qPCR-based ChIP studies, High Five insect cells (Thermo Fisher Scientific, USA) were infected with an HIF-3α2 expression plasmid containing the FLAG-His tag. The denatured HIF-3α2 protein was then purified by QIAexpress metal chelate chromatography (QIAGEN, Germany) according to manufacturer’s instructions. Polyclonal antisera were produced at Innovagen Ab (Lund, Sweden) by immunizing rabbits with the denaturated recombinant HIF-3α2 protein. The antisera were tested to exclude cross-reactivity with HIF-1α and HIF-2α, after which the HIF-3α2 antibody was purified using HiTrap Protein G HP columns (Amersham, USA) according to manufacturer’s instructions.

### cDNA microarray and qPCR analysis

For overexpression, 300 000 Hep3B cells were co-transfected once with either 1000 ng of pcDNA3.1/Zeo(-) or 1000 ng of HIF-3α2 plasmid with 1000 ng of HIF-β plasmid using FuGENE HD (Promega) and cultured for 24 h in normoxia and then 24 h in 1% hypoxia. For RNA interference, 300,000 cells were transfected twice with *HIF3A* siRNA (siGENOME, USA, MQ-010068-03-0005) targeting all *HIF3A* splice variants using siPORT NeoFX (Ambion, USA) at a 24-h interval and cultured under hypoxia for 24 h after the second transfection. All samples were prepared in triplicate and pooled for cDNA microarray analysis. RNA was isolated by E.Z.N.A Total RNA kit I (Promega). cDNA was prepared using the iScript cDNA Synthesis Kit (Bio-Rad, USA). The microarray was conducted using two Affymetrix Human Genome U133 Plus 2.0 Array chips with an Affymetrix Gene Chip Scanner 3000 7G (Thermo Fisher Scientific, USA) in Biocenter Oulu DNA Analysis Core, and the data were analyzed using the Chipster software (https://chipster.csc.fi/, version 3.12) [33]. Functional pathway analyses were carried out by Chipster using hypergeometric test for Gene Ontology (GO) with default settings. The microarray data have been deposited in the Gene Expression Omnibus database with accession number GSE128847.

The results of the microarray analysis were verified by quantitative real-time PCR (qPCR) with gene-specific primers (Table 1) and SsoFast EvaGreen Supermix (Bio-Rad) with a CFX96 Touch real-time PCR detection system (Bio-Rad). TATA box-binding protein (*TBP*), or β-actin (*ACTB*) and hypoxanthine phosphoribosyltransferase 1 (*HPRT1*) mRNA were used as reference genes for Hep3B and SK-N-AS cells, respectively.

### Chromatin immunoprecipitation (ChIP) followed by high-throughput sequencing (ChIP-seq)

ChIP experiments were performed as described previously [34]. Briefly, 1.6 × 10^6^ Hep3B cells were co-transfected with 6 000 ng of HIF-3α2-V5 and 4000 ng of HIF-β plasmids, cultured under normoxic conditions for 24 h and continued 24 h at 1% oxygen prior to ChIP. For the qPCR based ChIP studies, the control samples were co-transfected with 10,000 ng of pcDNA3.1-V5-HisA. Cells were crosslinked with 1% (v/v) formaldehyde and harvested for sonication to an average fragment size of 200–400 bp using Bioruptor UCD-300-TO (Diagenode, USA). The chromatin was immunoprecipitated for ChIP-seq with V5-tag antibody (R960-25, Invitrogen, USA) and for qPCR-based ChIP assays with the HIF3A antibody described above, and normal rabbit IgG (sc-2027, Santa Cruz Biotechnology, USA). ChIP-seq samples were processed according to Illumina’s instructions and DNA libraries were sequenced using Illumina HiSeq System (Illumina, USA) in the EMBL Gene Core Facility (Heidelberg, Germany). The qPCR-based ChIP results were normalized with respect to input. Fold changes were calculated using the formula 2^−(ΔCt)^, where ΔCt is Ct_(immunoprecipitated DNA)_ − Ct_(input)_ and Ct is the cycle at which the threshold line is crossed. The primers are listed in Table 1. The ChIP-seq data have been deposited in the Gene Expression Omnibus database with accession number GSE129491.

### HIF-3α2 peak calling and Integrative Genomics Viewer visualization

The original Fastq files were trimmed by ngsShoRT with the option “lqr_5adpt_tera”. Reads were aligned to hg19 human genome by Bowtie2. For the functional analyses, peaks were called by Homer with default parameters. De novo motifs were discovered by Homer. To visualize HIF-3α2 binding at certain target genes, peaks were called using the MACS algorithm with input as control. The bedGraph format from MACS was converted to bigwig using the UCSC pre-compiled utilities bedGraphToBigWig provided with chromosome sizes. Finally, the bigWigToWig utility was used to produce Wig files that were converted to TDF files for Integrative Genomics Viewer (IGV) visualization. The HRE location was searched by the “Find Motif” function in IGV with pattern “RCGTG”.

### HIF-3α overexpression and knockdown experiments and EPO-ELISA

For HIF-α overexpression EPO-ELISA experiments, 140,000 Hep3B cells were transfected with 1 200 ng of either pcDNA3.1-V5-HisA, HIF-3α2-V5, HIF-1α or HIF-2α, and 1000 ng of HIF-β plasmids, using FuGENE HD. For SK-N-AS cells, 250 000 cells were transfected with 900 ng of either pcDNA3.1-V5-HisA, HIF-3α2-V5, HIF-1α or HIF-2α, and HIF-β plasmids using FuGENE HD. For knockdown experiments in the SK-N-AS neuroblastoma cell line, the cells were seeded at 250,000 cells per well and transfected with *HIF3A* siRNA SR312024B (OriGene, USA) twice at an interval of 24 h using Lipofectamine RNAiMAX (Invitrogen). After the second transfection, the cells were cultured in 1% O_2_ for 24 h. Before isolating the total RNA as described above, the medium was collected and stored at –20 °C for EPO-ELISA. EPO-ELISA was carried out using the Quantikine IVD ELISA kit (R&D Systems, USA) according to manufacturer’s instructions. The absorbance was measured by Tecan Infinite m1000 PRO plate reader (Tecan, Austria) using 450 nm as the primary wavelength and 600 nm as the reference wavelength.

### Dual luciferase reporter assay

45,000 ChoK1 cells were co-transfected once with 200 ng of wild-type EPO, ANGPTL4, HIST1H2BK, or mutated HIST1H2BK luciferase reporters with HIF-1α, HIF-2α, or HIF-3α2-V5 plasmids at one (100 ng) or two concentrations (100 ng and 300 ng) as indicated, with or without 200 ng of the HIF-β overexpression plasmid. The empty pcDNA3.1 vector was used to balance the amount of transfected DNA. The pRL-CMV *Renilla* luciferase reporter was transfected for normalization at 10 ng. FuGENE HD (Promega) was used as transfection reagent. The cells were cultured under normoxic conditions for 24 h. The luciferase protein samples were prepared using the Dual-Luciferase Reporter Assay System (Promega) according to manufacturer’s instructions and analyzed using the Varioskan LUX plate reader (Thermo Fisher Scientific).

### Statistical analysis

Data are presented as means (± SD). Statistical analyses were carried out using the two-tailed Student’s *t* test using GraphPad Prism (version 7.03). Values of *p* < 0.05 are considered statistically significant, with * or # denoting *p* < 0.05, ** or ## *p* < 0.01, and *** or ### *p* < 0.001.

## Results

### Identification of HIF-3 target genes by microarray analysis

HIF-3α has previously been considered mainly as a dominant inhibitor of the hypoxia response by competitive binding of the other HIF-α subunits [5]. However, more recent studies have shown that the long human HIF-3 variants possess transactivation activity [9, 12, 30, 31, 35]. siRNA knockdown of all human HIF-3α variants simultaneously results in downregulation of certain hypoxia-responsive genes such as *EPO*, *GLUT1*, and *ANGPTL4*, and overexpression of long HIF-3α variants that possess the NTAD under conditions, where HIF-β is not limiting has an inducing effect on the same genes, with HIF-3α2 producing the most robust induction of *EPO* expression out of the five long HIF-3α variants [12]. To explore the potentially dual role of HIF-3 in the hypoxia response, we carried out a cDNA microarray screen of HIF-3α2 overexpression and siRNA knockdown of all *HIF3A* splice variants in hypoxic Hep3B cells.

**Fig. 1:**
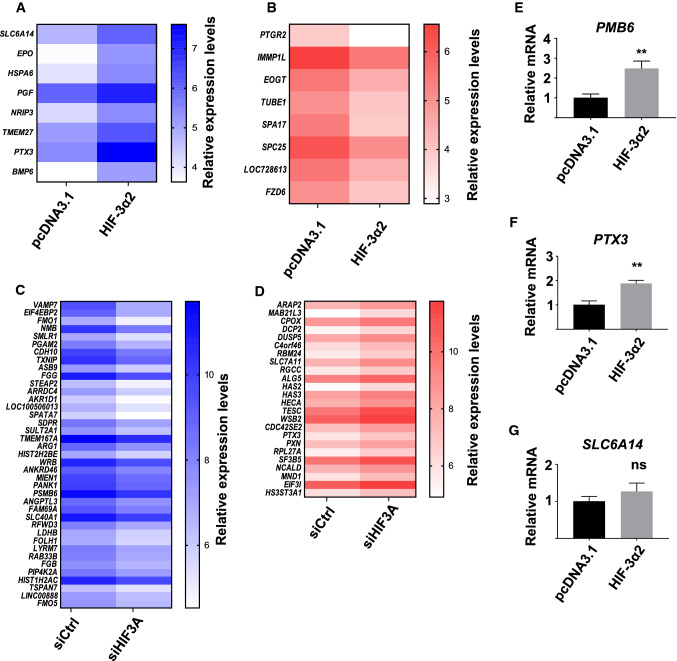
cDNA microarray screen of hypoxia-dependent HIF-3 target genes. The heatmaps show ≥ 2-fold upregulated (**a**) and downregulated (**b**) genes by HIF-3α2 and HIF-β co-overexpression, and downregulated (**c**) and upregulated (**d**) genes by *HIF3A* siRNA treatment in Hep3B cells incubated for 24 h in 1% hypoxia. The heatmaps are based on six pooled biological replicates on two microarray chips and show relative linear expression levels for control and treated cells as indicated. Darker shades of blue and red indicate higher levels of expression. **e**–**g** Validation of *BMP6*, *PTX3* and *SLC6A14* expression levels by qPCR with HIF-3α2 and HIF-β co-overexpression in Hep3B cells incubated in 1% hypoxia for 24 h. The qPCR data show upregulation of target genes that is in line with the microarray data. The mRNA levels are shown relative to *TBP* mRNA. Data are represented as means (± SD) from three independent experiments, *n* = 3. **p < 0.01, two-tailed Student’s *t* test

Setting the cut-off point of change in the expression level of a gene at ≥ 2-fold revealed eight upregulated (Fig. 1a) and eight downregulated (Fig. 1b) genes by HIF-3α2 and HIF-β co-overexpression in Hep3B cells incubated in 1% hypoxia for 24 h. The control cells were transfected with the empty pcDNA3.1/Zeo(−) vector and HIF-β. The upregulated genes include *EPO*, bone morphogenetic protein 6 (*BMP6*), pentraxin 3 (*PTX3*), and solute carrier family 6 member 14 (*SLC6A14*) among others. In contrast, the eight genes downregulated by HIF-3α2 overexpression include sperm autoantigenic protein 17 (*SPA17*) and frizzled family receptor 6 (*FZD6*), among others.

Next, treating Hep3B cells with either control siRNA or siRNA targeting all *HIF3A* splice variants and incubating the cells in 1% hypoxia for 24 h revealed a downregulation of 39 genes with ≥ 2-fold change (Fig. 1c). These genes include vesicle-associated membrane protein 7 (*VAMP7*), thioredoxin interacting protein (*TXNIP*), proteasome subunit type 6 (*PSMB6*), and angiopoietin-like 3 (*ANGPTL3*), among others. In comparison, 24 genes were upregulated ≥ 2-fold by siHIF3A knockdown (Fig. 1d), including tescalcin (*TESC*), solute carrier family 7 member 11 (*SLC7A11*), and eukaryotic translation initiation factor 3, subunit I (*EIF3I*).

**Fig. 2:**
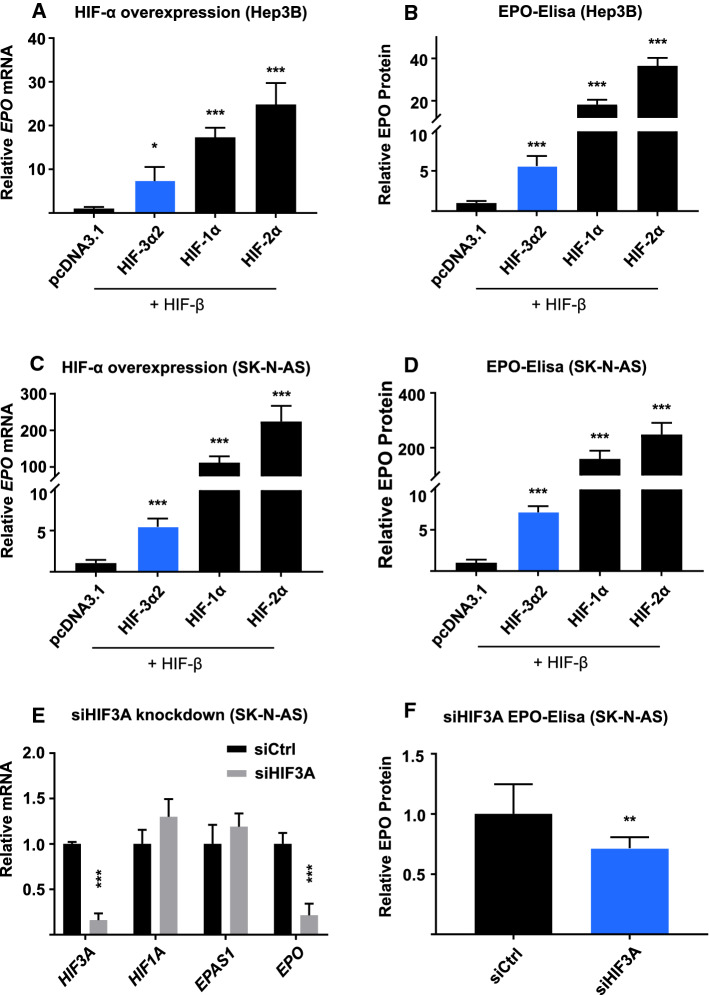
EPO regulation by HIF-3 in two cell lines. *EPO* mRNA and protein levels are upregulated by HIF-α overexpression in Hep3B (**a**, **b**) and SK-N-AS cells (**c**, **d**) when co-transfected with HIF-β. Treating SK-N-AS cells with siRNA targeting all *HIF3A* variants results in statistically significant downregulation of *EPO* mRNA and protein levels (**e**, **f**). Fold changes are relative to cells co-transfected with empty pcDNA3.1-V5-HisA vector and HIF-β, or control siRNA. *EPO* mRNA levels are relative to *TBP* for Hep3B cells, and *ACTB* and *HPRT1* for SK-N-AS cells. Data represent means (± SD) from three independent experiments, *n* = 6–9. *p < 0.05, **p < 0.01, ***p < 0.001, two-tailed Student’s *t* test

The cDNA microarray data suggest that HIF-3 has both inductive and inhibitory effects on global gene expression. As the microarray analysis setup was designed to provide an initial screen of the effects of HIF-3 on gene expression, it should only be taken as indicative. To verify the changes observed by HIF-3α2 and HIF-β co-overexpression on cDNA microarray, the upregulation of a subset of genes was confirmed by qPCR. HIF-3α2 overexpression produces an upregulation of *BMP6* (2.5 (± 0.37)-fold, Fig. 1e) and *PTX3* (1.9 (± 0.13)-fold, Fig. 1f) but not of *SLC6A14* (Fig. 1g). Similarly, *EPO* expression was validated on mRNA and protein levels (Fig. 2). Of note, *EPO* is expressed at a low level in Hep3B cells and thus only appears on the HIF-3α2 overexpression microarray.

Finally, functional enrichment analyses were carried out with Chipster [33]. Setting the cut-off point at 66% upregulation by HIF-3α2 and HIF-β co-overexpression revealed an upregulation of 59 genes. GO enrichment analyses suggest that HIF-3α2 is involved in DNA replication-dependent nucleosome assembly, vascular endothelial growth factor receptor signaling pathway, urogenital system development, and erythrocyte homeostasis (Table 2). A cut-off point of downregulation by 40% upon siHIF3A treatment revealed 126 downregulated genes. GO analysis shows enrichment of genes involved in heterotypic cell–cell adhesion, plasminogen activation, negative regulation of endothelial cell apoptotic process, blood coagulation, and fibrin clot formation as well as fibrinolysis (Table 3).

**Table 2:** Gene ontology (GO) functional analysis of upregulated genes by HIF-3α2 overexpression in Hep3B cells

GO term	*p* value	Description
GO:0072163	0.00042	Mesonephric epithelium development
GO:0033189	0.00193	Response to vitamin A
GO:0006335	0.00283	DNA replication-dependent nucleosome assembly
GO:0048010	0.00295	Vascular endothelial growth factor receptor signaling pathway
GO:0055093	0.00389	Response to hyperoxemia
GO:0000188	0.00418	Inactivation of MAPK activity
GO:0001655	0.00458	Urogenital system development
GO:0034101	0.00554	Erythrocyte hemostasis
GO:0001657	0.00571	Ureteric bud development
GO:0032094	0.0061	Response to food
GO:0032094	0.00648	Positive regulation of bone mineralization
GO:1901532	0.00722	Regulation of hematopoietic progenitor cell differentiation

**Table 3:** Gene Ontology (GO) functional analysis of downregulated genes by siHIF3A treatment of Hep3B cells

GO term	*p* value	Description
GO:0034116	0.00019	Positive regulation of heterotypic cell–cell adhesion
GO:0045921	0.00042	Positive regulation of exocytosis
GO:0043436	0.00066	Oxoacid metabolic process
GO:0031639	0.00079	Plasminogen activation
GO:1902042	0.00096	Negative regulation of extrinsic apoptotic signaling pathway via death domain receptors
GO:2000352	0.00115	Negative regulation of endothelial apoptotic process
GO:0072378	0.0124	Blood coagulation, fibrin clot formation
GO:0042730	0.00145	Fibrinolysis
GO:0051592	0.00152	Response to calcium ion
GO:1900026	0.00156	Positive regulation of substrate adhesion-dependent cell spreading

### The expression of EPO correlates with the knockdown of HIF3A and overexpression of HIF-3α2

As the microarray data indicate upregulation of EPO by HIF-3α2 overexpression (Fig. 1a), and as we have previously shown that siRNA knockdown of all *HIF3A* splice variants results in downregulation of *EPO* mRNA by 39–60% and protein by 28–73% in Hep3B cells [12], we carried out further HIF-3α2 overexpression experiments in Hep3B and the EPO-producing SK-N-AS neuroblastoma cells, as well as siHIF3A knockdown experiments in the SK-N-AS cells. *EPO* mRNA was upregulated 7- and 5-fold in Hep3B and SK-N-AS cells, respectively, with HIF-3α2 overexpression (Fig. 2a, c). In comparison, overexpression of HIF-1α and HIF-2α resulted in 17- and 25-fold upregulation of *EPO* mRNA in Hep3B cells, and in 110- and 220-fold upregulation in SK-N-AS cells, respectively (Fig. 2a, c). A 79% downregulation of *EPO* mRNA level was observed in SK-N-AS cells with siHIF3A treatment (Fig. 2e). It is highly unlikely that this was due to non-specific knockdown of *HIF1A* or *HIF2A,* as their mRNA levels were unchanged (Fig. 2e). Of note, previous experiments using siRNA targeting *HIF1A* mRNA in Hep3B cells, Kelly neuroblastoma cells, and cortical astrocytes have shown no effect on *EPO* expression [23, 27].

Changes in *EPO* expression were then analyzed at protein level by ELISA. Overexpression of HIF-3α2, HIF-1α, or HIF-2α resulted in sevenfold, 160-fold and 250-fold increases in EPO protein level in SK-N-AS cells, respectively (Fig. 2d). In Hep3B cells, similar treatment resulted in sixfold, 18-fold and 36-fold changes, respectively (Fig. 2b). Treating SK-N-AS cells with siRNA targeting all *HIF3A* splice variants resulted in downregulation of EPO protein level by 28% (Fig. 2f), which is in line with previous results obtained in Hep3B cells [12].

**Fig. 3:**
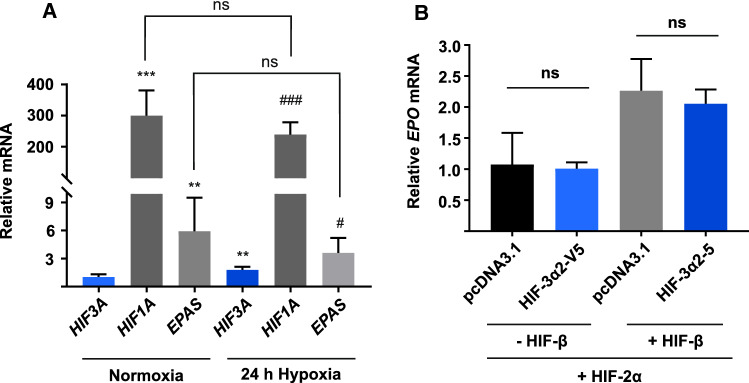
Hypoxic Hep3B cells express HIF-2α and HIF-3α at considerably lower levels than HIF-1α. **a** Hep3B cells were incubated in normoxia (pO_2_ 21%) and 1% hypoxia for 24 h before isolation of mRNA and quantification by qPCR. HIF-1α mRNA is 240 to 300-fold more abundant than HIF-3α mRNA, while HIF-2α mRNA abundance is only 2-fold higher than that of HIF-3α in hypoxia. HIF-3α expression is induced by hypoxia. Data are represented as means (± SD) from three independent experiments, *n* = 6. **p < 0.01, ***p < 0.001 against *HIF3A* mRNA abundance in normoxia, ^#^*p* < 0.05, ^###^*p* < 0.001 against *HIF3A* mRNA abundance in 1% hypoxia, two-tailed Student’s *t* test. **b** Hep3B cells were transfected with the HIF-2α overexpression plasmid together with either the empty pcDNA3.1 vector or the HIF-3α2 overexpression plasmid, and with or without the HIF-β overexpression plasmid as indicated, and incubated in 1% hypoxia for 24 h. Co-overexpression of HIF-3α2 does not induce or inhibit *EPO* expression upon HIF-2α overexpression. However, co-overexpression of HIF-β doubles *EPO* mRNA abundance. Data are represented as means (± SD) from three independent experiments, *n* = 3. *p < 0.05, **p < 0.01, two-tailed Student’s *t* test

Next, to assess whether *HIF3A* mRNA is expressed at a biologically relevant level, we compared the mRNA abundances of the three HIF-α isoforms by qPCR in normoxic Hep3B cells and Hep3B cells that were incubated in 1% hypoxia for 24 h. As expected, the *HIF3A* mRNA is induced by 73% in hypoxia (Fig. 3a). Previous studies have shown that this hypoxic induction is HIF-1 dependent [9, 10]. In both normoxic and hypoxic Hep3B cells, HIF-1α is the predominant HIF-α isoform with a 240–300-fold abundance over *HIF3A* mRNA (Fig. 3a). However, hypoxic Hep3B cells express HIF-3α and HIF-2α mRNA at a 1:2 ratio (Fig. 3a). The data—especially that of *HIF1A* expression—are to be treated with caution as differences in primer efficiencies cannot be excluded.

Finally, to explore whether co-overexpression of HIF-2α and HIF-3α2 with or without HIF-β alters *EPO* expression, we transfected Hep3B cells with the HIF-α and HIF-β overexpression plasmids as indicated and measured *EPO* mRNA abundances by qPCR (Fig. 3b). Of note, the pcDNA3.1/Zeo(−) backbone produces an upregulation of HIF-α mRNA by a fold of a few hundred, which most likely saturates *EPO* expression. No statistically significant changes were observed in *EPO* expression with or without HIF-β upon co-overexpression of HIF-2α and HIF-3α2 (Fig. 3b). Based on these findings, it is unlikely that HIF-3α2 acts as a dominant inhibitor of HIF-2α in the context of *EPO* expression.

### The long HIF-3 αβ dimer binds target gene promoters via the canonical HRE

**Fig. 4:**
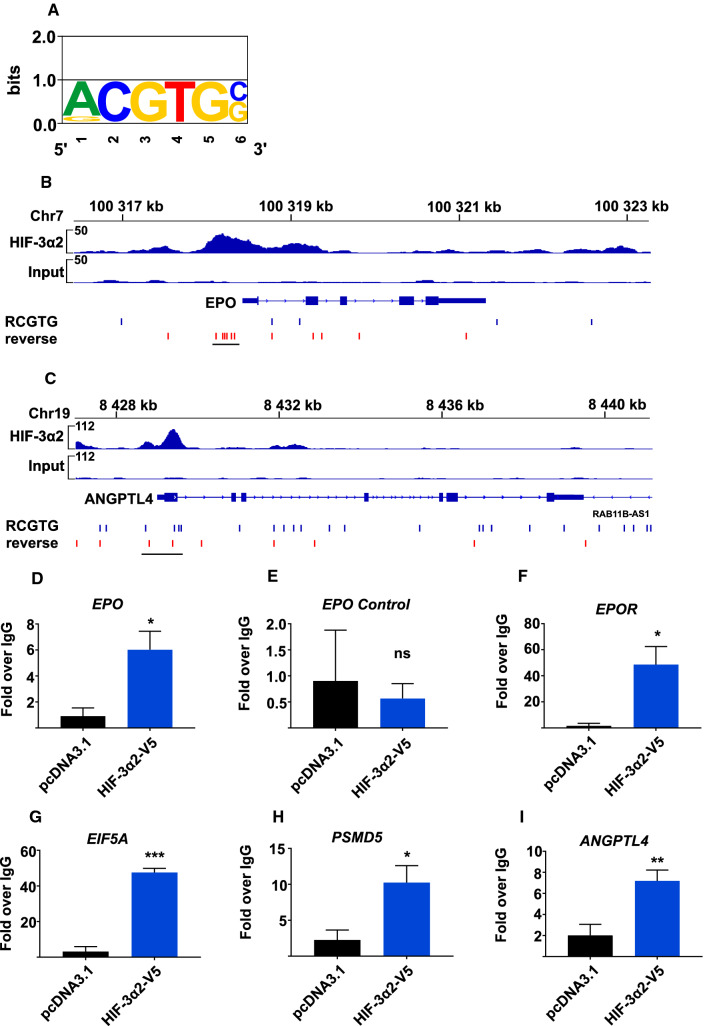
HIF-3 binding is associated with the canonical HRE in the promoter regions of its target genes. **a** HIF-3α2 ChIP-seq enrichment signal associates with the canonical HRE sequence with preference for A at position R. **b**, **c** HIF-3α2 enrichment is observed on the *EPO* and *ANGPTL4* genes near the promoter-TSS, co-localizing with sites that contain six canonical HRE sequences (5′-RCGTG-3′, included HREs underlined) on forward and reverse strands as denoted by blue and red lines, respectively. Interestingly, no HIF-3α2 enrichment is detected on the LIE immediately 3′ to *EPO*. HIF-3α2 enrichment (first track) is shown relative to input (lower track). Samples were prepared in triplicate and pooled for ChIP. **d**–**i** Validation of a subset of HIF-3α2 chromatin-binding enrichment sites by ChIP-qPCR. ChIP-qPCR by HIF3A and normal rabbit IgG antibodies shows amplification in the HIF-3α2 enrichment site on five genes, namely *EPO* (**d**), *EPOR* (**f**), *EIF5A* (**g**), *PSMD5* (**h**), and *ANGPTL4* (**i**), and no amplification with a control primer set designed to target regions of *EPO* where no enrichment is observed (**e**), *n* = 3. The ChIP-qPCR results are normalized with respect to input. *p < 0.05, **p < 0.01, ***p < 0.001, two-tailed Student’s *t* test

To analyze HIF-3 binding to its target genes, we performed ChIP-seq analysis after HIF-3α2 and HIF-β co-overexpression in Hep3B cells. De novo motif analysis of the peaks shows that the most significant motif for HIF-3α2 enrichment is the canonical HRE core sequence, 5′-RCGTG-3′, with the R position showing preference for adenine (Fig. 4a). The motif is shared most significantly by HIF-β. Overall, the data show that the HIF-3α2 peaks are enriched in the promoter regions (data not shown, GSE129491).

Next, ChIP-seq shows enrichment for HIF-3α2 in the promoter-TSS within the 0.4-kb 5′-flanking region of *EPO*, and some enrichment 3′ end to intron 1 (Fig. 4b), a region involved in liver-specific expression [19, 20]. Enrichment is not evident immediately at 3′ end of *EPO* on the 256-bp LIE (Fig. 4b) which has been identified as a crucial site for HIF-2, but not HIF-1, driven *EPO* regulation in the liver [20, 22, 23]. The 5′-flanking enrichment site contains six canonical HREs on the reverse strand, showing HIF-3α2 binding mainly across the first four HREs. In contrast, the LIE contains only a single HRE. Although the *ANGPTL4* gene does not show up on the cDNA microarray data, previous studies have shown that its hypoxic induction is HIF-3 dependent [9, 12], and thus, we decided to pursue it further on ChIP-seq. Two chromatin regions of enrichment for HIF-3α2 are detected near the promoter-TSS of *ANGPTL4*, 290 bp upstream and 380 bp downstream from the TSS (Fig. 4c). The downstream HIF-3α2-enriched region contains up to four canonical HREs.

Finally, we validated the ChIP-seq data by ChIP-qPCR with primer sets designed to span the HIF-3 binding regions. ChIP-qPCR shows relative enrichment of HIF-3α2 occupancy over IgG using primer sets for *EPO* [6.0 (± 1.4)-fold], *EPOR* [48.6 (± 13.9)-fold], *EIF5A* [47.5 (± 2.4)-fold], *PSMD5* [10.2 (± 2.3)-fold], and *ANGPTL4* [7.2 (± 1.0)-fold], and no enrichment over IgG with a primer set designed to target a region of *EPO,* where no HIF-3 binding was observed (Fig. 4d–i).

### HIF-3α2 overexpression may result in frivolous HRE-dependent chromatin binding

**Fig. 5:**
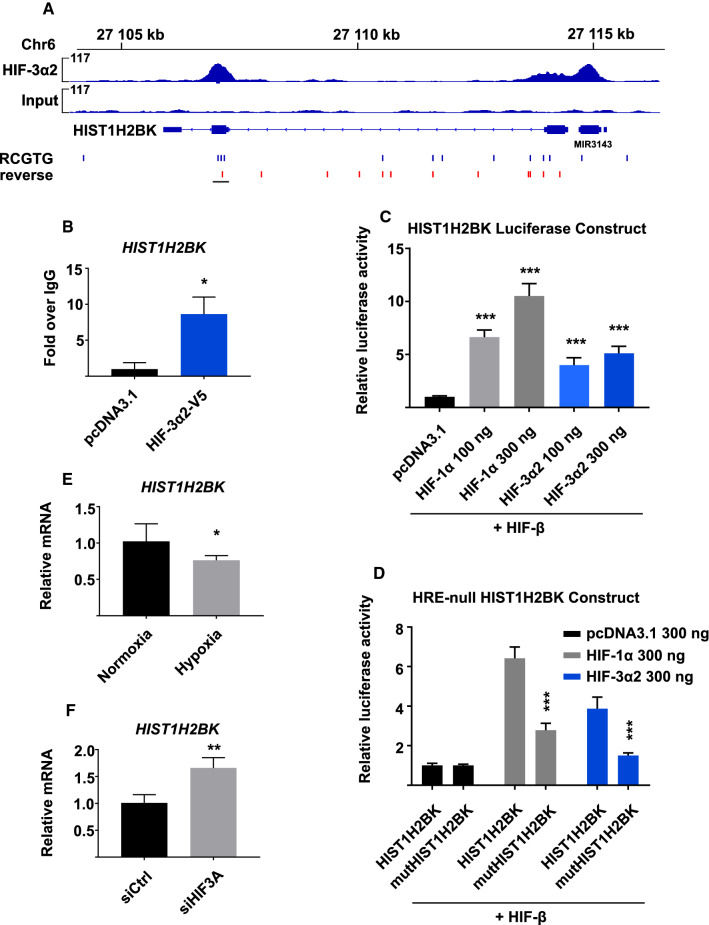
HIF-3α2 overexpression may result in frivolous HRE-dependent chromatin binding. **a** ChIP-seq shows HIF-3α2 enrichment on the *HIST1H2BK* gene near the promoter-TSS, co-localizing with a site that contains four canonical HRE sequences. **b** ChIP-qPCR confirmation of HIF-3α2 binding on *HIST1H2BK*. **c** Luciferase reporter construct containing the HIF-3α2 enrichment site on *HIST1H2BK* shows statistically significant upregulation by HIF-1α and HIF-3α2. Induction by HIF-3α2 plateaus already at the lower 100 ng transfection dosage. The data represent means (± SD) from three independent experiments, *n* = 8–9. **d** Four HREs observed in the HIF-3α2 enrichment site on *HIST1H2BK* were mutated to 5′-ATTTA-3′ to study the HRE-dependency of HIF-3α2 and HIF-1α binding, showing 66–69% decrease in luminescence signal. The data represent means (± SD) from four independent experiments, *n* = 11–12. **e** Hep3B cells were incubated in normoxia (pO_2_ 21%) and 1% hypoxia to study the hypoxia-dependent expression of *HIST1H2BK*. Hypoxia downregulates *HIST1H2BK* expression by 25%, representing means (± SD) from three independent experiments, *n* = 6. **f** Hep3B cells were treated with control siRNA or siRNA targeting all splice variants of the *HIF3A* locus, and incubated in 1% hypoxia for 24 h. *HIF3A* knockdown upregulates *HIST1H2BK* expression by 65%. The data represent means (± SD) from three independent experiments, *n* = 3. *p < 0.05, **p < 0.01, ***p < 0.001, two-tailed Student’s *t* test

To further explore the chromatin binding capacity of the long HIF-3 αβ dimer, we focused on Histone Cluster 1 H2B Family Member K (*HIST1H2BK*) as the ChIP-seq data suggest HIF-3α2 association with this gene. The ChIP-seq analysis revealed an enrichment of HIF-3α2 occupancy in the promoter region 440 bp upstream from the TSS of *HIST1H2BK* (Fig. 5a). This region contains four canonical HREs. ChIP-qPCR confirmed HIF-3α2 binding with a 7.7 (± 2.6)-fold relative enrichment over IgG (Fig. 5b).

To validate the activity of the *HIST1H2BK* regulatory sequence, we cloned the HIF-3α2-bound genomic region of *HIST1H2BK* in front of a luciferase gene in the pGL4.75 vector. The reporter plasmid was co-transfected into ChoK1 cells with either HIF-1α or HIF-3α2 at two concentrations (100 ng or 300 ng) with HIF-β and the *Renilla* reporter for normalization, after which the ChoK1 cells were incubated in normoxia for 24 h. Both HIF-1α and HIF-3α2 co-overexpressed with HIF-β induce the transcription of the luciferase reporter, with HIF-1α producing higher levels of luminescence signal in a dose-dependent manner (Fig. 5c). HIF-3α2 reaches a plateau already at the lower plasmid concentration (Fig. 5c). To study the HRE-dependency for HIF-1α and HIF-3α2 binding, we mutated the four HREs in the *HIST1H2BK* luciferase reporter to 5′-ATTTA-3′. The luminescence signal produced by the mutated *HIST1H2BK* luciferase reporter with HIF-1α and HIF-3α2 overexpression was reduced by 66% and 69%, respectively, providing further evidence that the HREs are necessary for maximal transcription and are the main but not sole determinant of HIF binding (Fig. 5d).

However, functional studies suggest that the endogenous HIF-3 αβ dimer may not bind the *HIST1H2BK* gene. We quantified the *HIST1H2BK* mRNA levels in normoxic and hypoxic Hep3B cells after incubation in 1% hypoxia for 24 h. Hypoxia downregulates *HIST1H2BK* expression by 25% in Hep3B cells (Fig. 5e). Finally, treating Hep3B cells with control siRNA or siRNA targeting all *HIF3A* splice variants and incubating the cells in 1% hypoxia for 24 h shows that *HIF3A* silencing produces an upregulation of *HIST1H2BK* by 65% (Fig. 5f). Therefore, it is possible that some of the HIF-3α2 occupancy observed on ChIP-seq is a result of overexpression and may not reflect endogenous function highlighting the importance of validation of the data at an endogenous level.

### Synergistic activity at the EPO promoter with HIF-3α2 and HIF-1α or HIF-2α co-overexpression

**Fig. 6:**
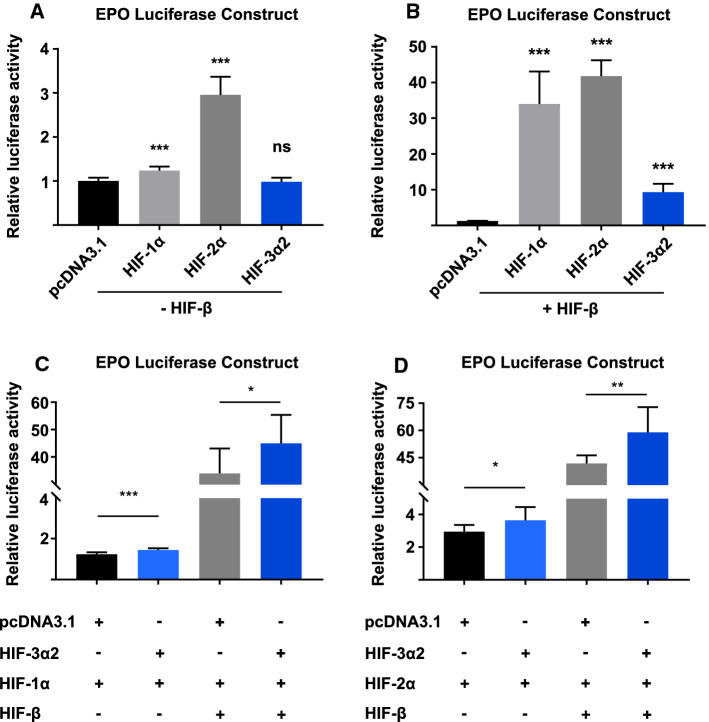
Synergistic induction of the *EPO* luciferase reporter by co-overexpression of HIF-1α or HIF-2α with HIF-3α2. **a**, **b** ChoK1 cells were transfected with the *EPO* luciferase reporter containing the HIF-3α2 binding site near the *EPO* promoter-TSS and either the HIF-1α, HIF-2α, or HIF-3α2 overexpression plasmid with or without HIF-β in normoxia. All three HIF-α isoforms can induce the activity of the *EPO* luciferase reporter when co-overexpressed with HIF-β. HIF-2α produces the most robust upregulation of the reporter. HIF-β significantly enhances the transactivation capacity of all HIF-α isoforms. The data represent means (± SD) from three independent experiments, *n* = 9. **c**, **d** Co-overexpression of HIF-1α or HIF-2α with HIF-3α2 produces a synergistic upregulation of the *EPO* reporter, representing means (± SD) from three independent experiments, *n* = 9. The relative luciferase activity is normalized against the pRL-CMV *Renilla* reporter. *p < 0.05, **p < 0.01, ***p < 0.001, two-tailed Student’s *t* test

In contrast to the *HIST1H2BK* gene, functional experiments in the Hep3B and SK-N-AS cell lines suggest that the long HIF-3 αβ dimer is involved in the regulation of *EPO* expression, whereas ChIP assays demonstrate that this regulation is via direct chromatin binding. To validate the activity of the regulatory sequence in the *EPO* promoter, we cloned the HIF-3α2-bound genomic region into the pGL4.75 luciferase vector. The *EPO* luciferase reporter was then co-transfected into ChoK1 cells with one of the HIF-α isoform overexpression plasmids, the *Renilla* reporter for normalization, and either the empty pcDNA3.1/Zeo(−) vector or the HIF-β overexpression plasmid. Next, the cells were incubated in normoxia for 24 h. Both HIF-1α and HIF-2α can induce the transcription of the luciferase reporter without HIF-β, with HIF-1α producing a 20% increase and HIF-2α producing up to a 3 (± 0.41)-fold increase over the control cells (Fig. 6a). With a co-overexpression of HIF-β, all HIF-α isoforms can induce the *EPO* luciferase reporter by 34.2 (± 9.1)-fold, 41.6 (± 4.5)-fold, and 9.0 (± 2.3)-fold for HIF-1α, HIF-2α, and HIF-3α2, respectively (Fig. 6b).

Finally, to further explore the transactivation capacity of HIF-3α2, we co-overexpressed HIF-3α2 with HIF-1α or HIF-2α and measured the relative luciferase activity produced by the *EPO* luciferase reporter. Without HIF-β, HIF-3α2 induced the relative luciferase activity by 17% and 21% over HIF-1α or HIF-2α alone, respectively (Fig. 6c, d). With HIF-β, however, a co-overexpression of HIF-1α or HIF-2α with HIF-3α2 produced a 45.6 (± 10.4)-fold and 59.6 (± 14.0)-fold increase in the relative luciferase activity meaning that HIF-3α2 induced the luciferase activity by 34% and 44% over HIF-1α or HIF-2α alone, respectively (Fig. 6c, d).

## Discussion

The contributions of HIF-3 and especially those of the long HIF-3α splicing variants to the regulation of the hypoxia response are yet largely unknown. We set out here to study the transactivation capacity of HIF-3α2 in more detail. Among the long HIF-3α variants, HIF-3α2 has been previously shown to induce the highest upregulation of *EPO* mRNA in Hep3B cells; HIF-3α2 is also expressed in the fetal and adult liver and kidney, which are the main EPO producing tissues [10, 12]. We explored HIF-3 target genes by cDNA microarray analysis in Hep3B cells and concluded that eight genes were upregulated by ≥ 2-fold with HIF-3α2 overexpression and 39 genes were downregulated by ≥ 2-fold with siHIF3A treatment suggesting a HIF-3 specific transcriptional program. No overlap is observed between these microarray data, because the *HIF3A* siRNA result in a knockdown of all HIF-3α splice variants, while the overexpression experiments include only HIF-3α2. However, both knockdown and overexpression settings result in positive and negative effects on global gene expression suggesting that HIF-3 plays a dual role in the hypoxia response. In the context of overall hypoxia-dependent gene regulation, this can be considered a small subset of genes as HIF-1 and HIF-2 have been shown to regulate the transcription of over 1 500 human genes through direct transactivation according to different ChIP assays [14, 15, 36, 37]. In zebrafish, the overexpression of the long Hif-3 splice variant resulted in the upregulation of 136 unique genes, whereas Hif-1α overexpression upregulated up to 690 genes with 97 overlapping targets [30], supporting the view that HIF-3 is responsible for the oxygen-dependent regulation of a relatively small subset of hypoxia-inducible genes.

We validated the role of HIF-3 in regulating EPO signaling in vitro. We have previously shown that siRNA knockdown of all *HIF3A* splice variants simultaneously results in the downregulation of *EPO* mRNA in the Hep3B cell line [12] that was originally used to study the hypoxia-dependent regulation of *EPO* [19]. Here, we show using Hep3B and SK-N-AS, two cell lines capable of endogenous *EPO* production, that loss and overexpression of HIF-3 results in significant changes in *EPO* mRNA and protein levels, further indicating that HIF-3 has a role in erythropoiesis through *EPO* regulation. Of note, previous studies using siRNA to target *HIF1A* mRNA in similar cell lines have not produced significant effects in EPO expression [23, 27]. In the murine cardiomyocytes, the knockdown of *Hif3a* has been associated with a very minor upregulation of *EPO* expression, but an upregulation of HIF-1α and HIF-2α mRNA was also observed [38]. No human kidney-derived cell lines with inducible *EPO* expression exist, while the derivation of a murine kidney cell line with inducible *EPO* expression was reported only recently [39].

The last line of evidence supporting our hypothesis that HIF-3 is a transcription activator involved in the regulation of EPO signaling arises from ChIP and transactivation studies. In Hep3B cells, HIF-3α2 binds chromatin immediately 5′ to the *EPO* gene, and this genomic region is sufficient to transactivate a luciferase reporter construct by all three HIF-α isoforms. Interestingly, HIF-3α2 enrichment is not observed at the single HRE required for HIF-2 driven *EPO* regulation at the liver inducibility element immediately 3′ to *EPO* [17]. This may imply that HIF-3 does not compete for the binding sites used by HIF-1 and HIF-2 as was recently shown to be true between HIF-1 and HIF-2 despite a set of shared target genes [15, 40]. Finally, using the *HIST1H2BK* gene, we show that HIF-3 may redistribute to non-endogenous HREs upon overexpression and that chromatin occupancy studies should be paired with functional assays to dissect endogenous target genes.

Our data are in line with previous studies about the biological function of the long HIF-3 isoforms. We have shown that siRNA knockdown of all *HIF3A* splice variants and overexpression of certain long HIF-3 isoforms downregulate and upregulate, respectively, *EPO*, *ANGPTL4*, and *GLUT1*, but not *VEGF* which is predominantly a HIF-1 target [12]. Similarly, Zhang and colleagues characterized the role of Hif-3 as transcription activator in the zebrafish, and show that hypoxia and overexpression of human HIF-3α9 [10] induce *LC3C*, *REDD1* and *SQRDL* expression in human HEK293 and U2OS cell lines [30]. Furthermore, HIF-3α has been implicated in the progression of pancreatic cancer by directly binding the promoters of *RHOC* and *ROCK1* and transactivating the RhoC-ROCK1 pathway in cancer cells that overexpress *HIF3A* [41].

The data presented in this study may provide basis for human disease. Two single-nucleotide variants in the *HIF3A* locus have been associated with familial erythrocytosis [42]. The data reported here suggest that it may be through a direct transactivation effect. However, studies that are more sensitive (i.e., ChIP-seq using antibodies that recognize the endogenous HIF-3α protein, or RNA-seq to study the kinetics of *HIF3A* splicing and expression) are required to elucidate the role of HIF-3 further. Next, methylation of the *HIF3A* gene in blood cells and adipose tissue was recently shown to correlate with increased body-mass index in a genome-wide association study [43]. The data suggest that impairment of the HIF pathway may result in the dysregulation of body weight, which is in line with previous studies showing that HIF prolyl 4-hydroxylase-2 inhibition and hence HIF-α stabilization and activation of the hypoxia response pathway is protective of obesity and metabolic dysfunction [44, 45]. We have shown that demethylation increases *HIF3A* mRNA levels in Hep3B cells [10]. Here, we show that HIF-3α2 directly regulates the expression of *ANGPTL4*, which is involved in the induction of white adipose tissue lipolysis [46, 47] and may thus regulate energy metabolism. Other HIF-3 target genes identified by cDNA microarray, such as *ANGPTL3* and *PANK1*, may also be involved in metabolic regulation [48–51]. This link between HIF-3 and lipid metabolism may also explain why only a few cancer cell lines express the long *HIF3A* splice variants, whereas the HIF-1α that drives glucose metabolism is their major HIF form [10, 52]. However, further studies are required to elucidate the direct mechanisms of HIF-3 dependent regulation of body weight.

In conclusion, our present study shows that HIF-3 is involved in the regulation of a subset of hypoxia-inducible genes. According to our chromatin immunoprecipitation data, HIF-3 directly binds *EPO* in its promoter-TSS at a region harboring several HREs. This region is sufficient to drive the transcription of a luciferase reporter gene. We provide evidence that HIF-3 is a transcription factor required for maximal induction of *EPO* on mRNA and protein levels, and that HIF-3α2 is more likely to produce synergistic than inhibitory effects when co-overexpressed with HIF-1α or HIF-2α. We would, therefore, suggest that the next step in exploring the biological role of HIF-3 is to study murine *Hif3a* splice variants in *EPO* regulation in vivo, but it should be noted that splicing of the mouse Hif3a gene is far less complex than that of the human HIF3A gene [6, 7, 10, 53]. Therefore, inactivating the individual human *HIF3A* splice variants using the CRISPR-Cas9 technology or studying the kinetics of *HIF3A* and HIF-β expression through RNA-seq could be preferential approaches for future studies.
